# Meteorological Influence on Drinking Water Quality: Microbial Variability in Groundwater Wells and Piped Distribution Networks from Western Romania

**DOI:** 10.3390/microorganisms14010148

**Published:** 2026-01-09

**Authors:** Corneluta Fira-Mladinescu, Mădălin-Marius Margan, Roxana Margan, Florin Ardelean, Adrian Ioan Sînmârghițan, Delia Marincov, Ioana Tuță-Sas, Ioana Marin, Alexandru-Cristian Cîndrea, Diana-Alina Bodea, Sorina Maria Denisa Laitin

**Affiliations:** 1 Department of Microbiology, Discipline of Hygiene, Center for Studies in Preventive Medicine, Victor Babes University of Medicine and Pharmacy, 300041 Timisoara, Romania; fira-mladinescu.corneluta@umft.ro (C.F.-M.); tuta-sas.ioana@umft.ro (I.T.-S.); ioana.marin@umft.ro (I.M.); 2 Timiș County Public Health Directorate, 300029 Timisoara, Romania; florin.ardelean@dsptimis.ro (F.A.); sinmarghitan.adrian@dsptimis.ro (A.I.S.); delia.marincov@dsptimis.ro (D.M.); 3 Discipline of Public Health, Department of Functional Sciences, Victor Babes University of Medicine and Pharmacy, 300041 Timisoara, Romania; 4 Centre for Translational Research and Systems Medicine, Faculty of Medicine, Victor Babes University of Medicine and Pharmacy, 300041 Timisoara, Romania; 5 Doctoral School, Faculty of General Medicine, Victor Babes University of Medicine and Pharmacy Timisoara, 300041 Timisoara, Romania; alexandru.cindrea@umft.ro; 6 Emergency Clinical Municipal Hospital, 300254 Timisoara, Romania; 7 Doctoral School, Department of Geography, West University of Timisoara, 300209 Timisoara, Romania; diana.bodea96@e-uvt.ro; 8 Department XIII, Epidemiology University Clinic, Victor Babes University of Medicine and Pharmacy Timisoara, Eftimie Murgu Square 2, 300041 Timisoara, Romania; laitin.sorina@umft.ro; 9 Prevention Nosocomial Infections Department—CPCIN, Clinical Hospital of Infectious Diseases and Pulmonology, Gheorghe Adam Street 13, 300310 Timisoara, Romania

**Keywords:** drinking water quality, meteorological impact, groundwater wells, distribution networks

## Abstract

Climate variability plays a crucial role in shaping drinking water quality, yet the quantitative links between meteorological factors and microbiological contamination remain underexplored in temperate continental climates. A secondary data analysis was conducted on 15,394 microbiological water quality test results collected between 2015 and 2024, including heterotrophic plate counts (22 °C and 37 °C), coliform bacteria, and *Escherichia coli*, and their associations with local meteorological conditions across groundwater wells and piped distribution networks were examined. A clear distinction emerged: groundwater wells showed higher vulnerability to primary microbial contamination (coliforms and *E. coli*), whereas distribution networks exhibited higher odds of heterotrophic plate count exceedances, indicating greater susceptibility to post-treatment microbial regrowth. In groundwater wells, temperature showed strong positive associations with all microbial indicators (*p* < 0.001), with pronounced summer peaks in coliforms and *E. coli*, while precipitation triggered short-term contamination spikes characterized by a 2-day lag. In contrast, piped networks exhibited weaker and more delayed meteorological responses. These results highlight the need for a shift from climate-responsive to climate-pre-emptive water quality monitoring by incorporating meteorological forecasts, especially for non-chlorinated groundwater sources.

## 1. Introduction

Water quality is a critical determinant of public health, shaped by both natural and anthropogenic influences. Climate change and meteorological variability—including temperature fluctuations, precipitation patterns, and storm events—directly impact the microbial and chemical stability of both natural water reservoirs and urban drinking water supplies. Rainfall can introduce contaminants and affect turbidity, while also diluting chemical concentrations. Simultaneously, elevated temperatures promote bacterial proliferation and accelerate chemical transformations, posing further challenges to water safety and sustainability. Understanding these complex dynamics is essential for developing resilient and adaptive water management strategies [1,2,3].

Consequently, microbiological parameters play a central role in assessing the safety of drinking water. *Escherichia coli* (*E. coli*) remains the primary indicator of fecal contamination, while coliform bacteria indicate general hygiene and treatment efficacy [4,5]. Heterotrophic plate counts (HPC) at different temperatures provide insights into general bacterial populations and biofilm formation potential in distribution systems [6,7]. The European Union Drinking Water Directive (EU 2020/2184) mandates zero tolerance for *E. coli* and establishes strict limits for other microbiological parameters [8].

Beyond these immediate impacts, disruptive meteorological phenomena—such as heavy rainfall, droughts, and extreme temperature shifts—are increasingly linked to fluctuations in microbial water quality indicators, including *E. coli* and coliform bacteria. These effects frequently manifest with identifiable lag periods following weather events [9,10,11,12]. For instance, flash floods and prolonged droughts can lead to microbial contamination through surface runoff, sewer overflows, or the concentration of pollutants in groundwater. Long-term time-series analyses confirm that hydro-meteorological dynamics directly shape water quality trends, underscoring the need for more agile, weather-responsive monitoring protocols [13,14]. Despite growing evidence, empirical studies that integrate real-time meteorological data with microbiological assessments remain limited. Proactive, adaptive monitoring of drinking water quality is increasingly recognized as essential for safeguarding public health, especially under growing meteorological variability [15,16].

Recent advancements in IoT-based water quality monitoring systems have demonstrated significant improvements in early detection of contamination and operational efficiency, enabling timely interventions aligned with changing environmental conditions [17]. Open-source tools, such as WaterScope, and rapid field test kits further expand access to low-cost, on-site monitoring, particularly in underserved or climate-vulnerable regions [18]. These tools support community-based surveillance strategies, which increase coverage and empower frontline detection and mitigation efforts [19,20]. Systematic reviews also emphasize the benefits of integrating smart technologies, sensors, and artificial intelligence in water quality management, highlighting their role in improving adaptation to climate-related challenges [21,22].

To address these challenges, international frameworks such as the WHO’s guidelines on risk-based drinking-water surveillance advocate for protocols that adapt to local hazards, source characteristics, and meteorological triggers [23]. These flexible approaches, including Water Safety Plans, prioritize dynamic scheduling based on evidence of risk, moving beyond rigid compliance routines. Studies on smart water infrastructure also illustrate how integrating sensor networks with predictive analytics allows utilities and public health agencies to anticipate microbial risks, enhancing system resilience under climate change [24,25].

Together, this body of evidence underscores the importance of shifting from fixed monitoring schedules to adaptive protocols informed by real-time and weather-driven insights. Such transitions are vital for protecting drinking water safety, especially in regions like Western Romania—where climate variability intersects with vulnerable distribution systems. In this region, drinking water is commonly sourced either directly from individual or drilled groundwater wells—especially in rural communities—or delivered through piped distribution networks supplied by centralized groundwater abstractions in urban areas. These two supply types differ substantially in terms of exposure to environmental stressors, treatment practices, and vulnerability to microbial contamination. The coexistence of decentralized wells and piped distribution systems within a relatively homogeneous lowland hydrogeological setting makes Western Romania a uniquely suitable case study for investigating how meteorological variability influences microbiological drinking water quality. Understanding these interactions is crucial for developing effective water treatment strategies and ensuring public health protection in the context of increasing climate variability [26].

In addition to direct meteorological drivers, drinking water quality is strongly shaped by underlying structural and land-use vulnerabilities that modulate how climate signals translate into contamination risk. Groundwater supplies nearly half of the world’s drinking water, yet its susceptibility to contamination varies widely depending on hydrogeology, land use, sanitation coverage, and infrastructure condition. At a global scale, anthropogenic non-point source pressures—particularly intensive agriculture and inadequate sanitation—represent the most widespread threats to groundwater quality, especially in developing and transitional regions [27]. Agricultural nitrogen and pesticide inputs have led to persistent groundwater contamination across Europe, Asia, and Africa, with legacy pollutants often remaining for decades due to slow groundwater flow and limited natural attenuation [28,29]. In some regions, geogenic contaminants such as arsenic and fluoride further compound groundwater-related health risks.

Microbial groundwater contamination is closely linked to sanitation infrastructure, with poorly constructed or improperly sited pit latrines and septic systems facilitating fecal ingress into shallow aquifers, particularly in peri-urban and rural settings [30,31,32]. In parallel, aging piped distribution networks represent an additional contamination pathway, as leakage, pressure fluctuations, and structural deterioration allow external contaminants to infiltrate systems—vulnerabilities that are especially pronounced in transitional regions of Eastern Europe and are exacerbated by climatic wet–dry cycling [33]. Together, these non-climatic pressures interact with meteorological variability, shaping both baseline water quality and its sensitivity to weather extremes. Accounting for this interplay is essential for developing adaptive, risk-based drinking water monitoring and management strategies.

This study investigates how short- and medium-term meteorological variability influences microbiological drinking water quality in Western Romania, using a retrospective, decade-long dataset derived from routine regulatory monitoring.

The aim of this study is to quantify and compare the influence of meteorological variability on microbiological drinking water quality in groundwater wells and piped distribution networks in Western Romania. The purpose is to achieve a better understanding of when and why microbial risks increase and how weather-informed insights can be used to support more timely, adaptive, and preventive approaches to drinking water monitoring and protection.

## 2. Materials and Methods

This study represents a secondary data analysis of water quality laboratory results originally collected for regulatory and operational purposes between 2015 and 2024 in Timiș County by Public Health Directorate (DSP Timiș). Water samples were continuously submitted to the regional public health laboratory by public and private water supply operators, either in fulfilment of mandatory external audits under national drinking water legislation, or as part of voluntary internal monitoring programs. These submissions formed part of the National Drinking Water Monitoring Program coordinated by the Ministry of Health, as well as operator-initiated checks intended to ensure ongoing compliance or to investigate suspected water quality issues.

### 2.1. Study Area

**Figure 1:**
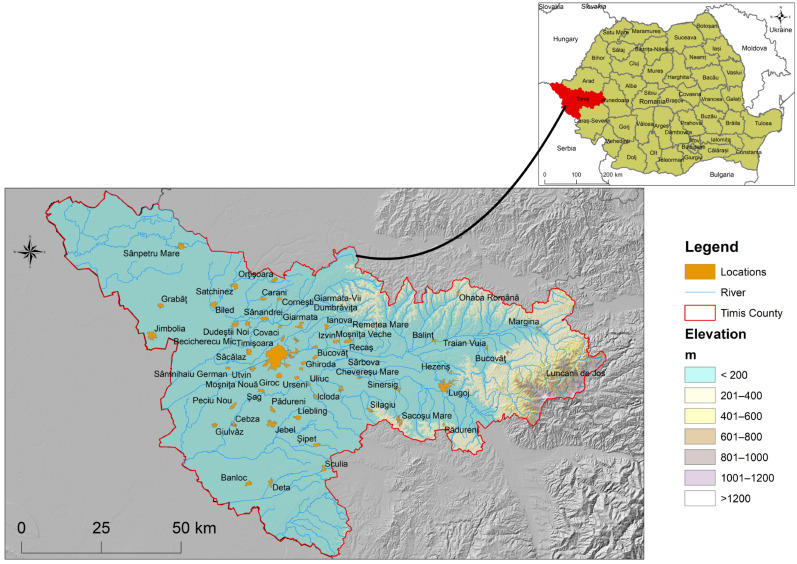
Spatial distribution of the monitoring locations across Timiș County, Western Romania, all sites being situated in lowland areas below 200 m elevation. The map illustrates the hydrographic network, county boundaries, localities, and elevation gradients relevant to the study area. The map was created using ArcGIS 10.4.1 software. Data sources include county boundaries, Romanian border, localities, and hydrographic network from geo-spatial.org, and the SRTM DEM at 30 m resolution from earthexplorer.usgs.gov.

The study was conducted in Timiș County, located in Western Romania (approx. 45°45’ N, 21°13’ E), covering an area of approximately 8697 km^2^. The county lies mostly within the Western Plain (Câmpia de Vest), with elevations below 200 m, as illustrated in Figure 1. The hydrographic network is dominated by the Bega and Timiș rivers, and the groundwater resources are primarily sustained by alluvial and phreatic aquifers in lowland areas [34,35].

The region experiences a temperate continental climate, with average annual temperatures between 10.5 °C and 11.5 °C, and annual precipitation ranging from 500 to 700 mm [36]. The study area focuses specifically on lowland zones, where all monitoring locations are situated (Figure 1), reflecting a relatively homogeneous hydrogeological and topographical context [34].

This area includes Timișoara Municipality (population 319,279), several smaller towns, and surrounding rural communities, with a total of around 450,000 inhabitants. While urban and small-town areas are primarily supplied through centralized piped distribution networks, many rural communities rely on drilled groundwater sources for drinking water, either via individual household wells or, less commonly, small community groundwater supply systems. These characteristics make the region particularly relevant for studying the impact of meteorological variability on groundwater quality in uniform plains settings [37].

### 2.2. Water Quality Data Collection

#### 2.2.1. Water Source Types

In this study, two primary drinking water supply types were analysed: Groundwater Wells are defined as individual or small-scale dug or drilled wells that draw directly from aquifers, typically shallow and unchlorinated. The small number of community-level water supply systems drawing from aquifers but subjected to centralized treatment were excluded from this category, as their management and risk profiles more closely align with treated distribution systems. Piped Distribution Networks are defined as centralized or semi-centralized distribution systems supplied primarily from surface water sources such as rivers, lakes, or reservoirs, and generally subjected to treatment (e.g., chlorination) before delivery to consumers.

#### 2.2.2. Sampling Locations

During the analysis of data collection patterns and metadata, we identified 53 distinct locations across Timiș County, Western Romania, between January 2015 and December 2024.

The lowland dominance in elevation, the proximity to major rivers, and the rural–peri-urban spread of sites collectively reflect the real-world configuration of water sources used by the population [38,39].

Although the majority of sampling sites were all located at an elevation of <200 m a.s.l., the underlying stratigraphy is vertically heterogeneous. Beneath the unconsolidated alluvial blanket (0–30 m, unconfined), semi-confined Quaternary sands and deeper confined Upper-Pannonian sand–sandstone aquifers occur. These distinct hydrogeological layers vary in their vulnerability to surface influences such as precipitation and contamination [28].

#### 2.2.3. Water Sampling Protocols

All water samples were collected by well owners or network system operators in accordance with national regulations and standardized procedures, following sampling instructions regularly distributed by DSP Timiș to ensure proper technique.

For bacteriological testing, water was collected in sterile bottles according to ISO 19458:2006 [40]. Samples were transported at ~4 °C to the laboratory the same day, accompanied by standardised forms detailing location, date, source type, requested analyses, and relevant sanitary or meteorological conditions to ensure traceability.

#### 2.2.4. Laboratory Analysis of Microbiological Parameters

All analyses were conducted in the accredited regional public health laboratory under routine quality-assurance protocols.

Microbiological quality was assessed using four key indicators: HPC 22 and HPC 37 (representing total germ counts at 22 °C and 37 °C, respectively), coliform bacteria and *E. coli*. Each parameter was categorized according to detection thresholds, with values classified as below the detection limit (e.g., <1/100 mL or 0/100 mL), detectable within the acceptable range, or above the detection limit (e.g., >100 CFU/mL or values explicitly marked “>300”). Binary exceedance variables were generated to quantify the proportion of samples surpassing recommended microbial limits.

Microbiological analyses were conducted using methods accredited by RENAR (Asociația de Acreditare din România—the Romanian Accreditation Association), in accordance with European standards and national regulations in effect at the time of testing. While core analytical principles remained consistent, the specific regulatory references evolved slightly over the study period. For example, ISO 9308-1 transitioned from the 2014 version to amendments A1:2017 and A1:2020 [41]. Most recently, Government Decision no. 7/2023 aligned microbiological standards with Directive (EU) 2020/2184, reflecting the latest European Union requirements for drinking water quality [8,42]. Table 1 summarizes the parameters, units, regulatory limits, and reference methods.

#### 2.2.5. Chlorination Practices in Piped Distribution Networks

All piped distribution networks included in this study are subject to routine disinfection by chlorination, in accordance with Romanian legislation (Government Decision No. 7/2023 transposing Directive (EU) 2020/2184) on the quality of water intended for human consumption. Chlorine dosing at treatment facilities is adjusted based on the quality of the raw water source, with the objective of achieving a free residual chlorine concentration of approximately 0.5 mg/L at the outlet of the treatment plant. Within the distribution network, residual free chlorine concentrations are typically maintained between 0.1 and 0.5 mg/L at distal points, as required by national regulations. Under certain operational or sanitary conditions, additional chlorination may be applied within the distribution system. However, along the transport pathway, chlorine residuals may decrease due to consumption processes such as reactions with organic matter, pipe materials, and biofilms. As a result, free or total chlorine concentrations may vary spatially and temporally within the network and at network endpoints.

Information on free or total chlorine residual concentrations was not systematically recorded in the routine laboratory reports used for this analysis and was therefore not available for inclusion as an analytical covariate. Consequently, the effects of chlorination intensity, temporal variability in disinfectant residuals, and potential temperature × chlorine interactions could not be directly evaluated in the statistical models. The observed differences between groundwater wells and piped distribution networks should thus be interpreted in the context of both meteorological influences and the presence of controlled disinfection practices, while acknowledging the absence of quantitative chlorine data as a study limitation. However, within the temperature ranges observed in this study, variations in ambient water temperature are not expected to substantially affect chlorine stability or disinfectant efficacy, as temperature-related degradation of free chlorine becomes operationally relevant primarily at much higher temperatures than those encountered in drinking water systems. Consequently, within the monitored range, temperature effects on microbial indicators should be interpreted as acting predominantly through microbial growth and environmental processes rather than through direct impairment of chlorination performance.

### 2.3. Water Quality Data Extraction and Integration Framework

#### 2.3.1. Automated PDF Parsing and Metadata Extraction

Each report included metadata such as the sampling date, geographical location, water source type (drilling or network)—classified using standardized coding systems.

The data was extracted using a custom Python version 3.12.0 (packages pdfplumber, tabula, re, pandas, tqdm, ProcessPoolExecutor) script to automate the parsing of these PDF reports. Given the heterogeneity in structure and formatting across documents, manual extraction was not feasible.

#### 2.3.2. Dataset Construction

**Figure 2:**
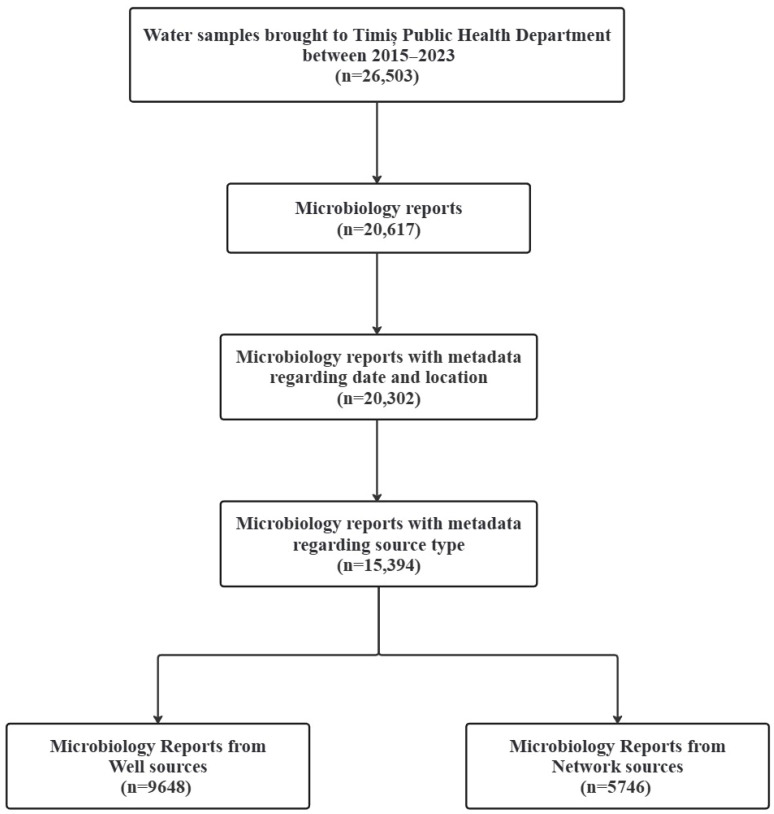
Flow diagram illustrating dataset construction of microbiology reports results by source type submitted to the Timiș Public Health Department between 2015 and 2024.

A total of 20,617 microbiology reports were retrieved during the study period. Based on presence of metadata clearly stipulating source type, 9468 microbiology reports were identified as belonging to groundwater wells and 5746 to network sources. The final database included 15,394 entries with complete metadata (date, location, and source type). It is important to note that a considerable number of reports lacked one or more results due to certain tests not being requested or performed for specific samples. A schematic overview of the entire data extraction and integration process is provided in Figure 2.

Prior to analysis, the dataset was screened for completeness and internal consistency. Records were checked for potential duplicates and implausible/outlier values; flagged entries were verified against available source metadata and excluded when needed.

### 2.4. Meteorological Data Acquisition and Processing

Meteorological variables were obtained from the Meteomanz automated monitoring network and included daily values—either averaged from hourly measurements for temperature (°C) and wind speed (m/s) or aggregated as daily totals for cumulative precipitation (mm) and total insolation (hours/day). The temporal framework was structured to capture diverse response dynamics between meteorological conditions and water quality, incorporating meteorological variables measured on the day of sampling as well as at lag intervals of 1, 2, 3, and 30 days. All meteorological variables were derived from a single centrally located station in Timișoara, considered representative for the study area due to its predominantly lowland topography and limited spatial extent, associated with minimal expected intra-regional climatic variability.

### 2.5. Statistical Analysis

Statistical analysis and graphical representation were conducted using R version 4.4.1, with the following packages: broom, dplyr, flextable, ggplot2, glmnet, knitr, lubridate, officer, purrr, stringr, tibble, tidyr, tidyverse, and viridis.

New variables to represent lagged meteorological parameters were generated and merged into the main dataset using a left join.

Descriptive statistics were employed to characterize water quality parameters and meteorological variables. The distribution of continuous variables was assessed through graphical methods, including histograms and quantile-quantile (Q-Q) plots, as well as by calculating skewness and kurtosis coefficients to evaluate symmetry and peakness. Continuous variables were summarized using the median and interquartile range (IQR), reflecting the non-parametric nature of the data. Categorical variables were described using absolute frequencies and corresponding percentages.

Univariate logistic regression models were employed to explore the association between key predictor variables and specific outcomes. Odds ratios (ORs) and corresponding 95% confidence intervals (CIs) were calculated to quantify the strength and direction of these associations.

Furthermore, to comprehensively evaluate the influence of meteorological variables on binary microbial outcomes, a Least Absolute Shrinkage and Selection Operator (LASSO) logistic regression was applied. All continuous predictor variables were standardized prior to model fitting to ensure comparability and appropriate penalization. The regularization parameter (λ), which controls the strength of the model penalty, was selected through cross-validation to optimize model performance.

Seasonal variability in microbial exceedance rates was additionally assessed using the non-parametric Kruskal–Wallis test.

Time series modelling was performed using Auto-Regressive Integrated Moving Average (ARIMA) and ARIMAX models. Model adequacy was assessed through Ljung–Box residual diagnostics, while predictive performance was quantified using root mean squared error (RMSE) and mean absolute error (MAE).

A *p*-value of less than 0.05 was regarded as statistically significant, with all tests evaluated using two-tailed criteria.

### 2.6. Data Limitations

A key limitation of the present study is the absence of precise well depth information for the majority of sampling locations. This restricts our ability to stratify groundwater quality results by aquifer depth or hydrogeological unit. Based on available source descriptions and typical rural infrastructure in Western Romania, the majority of monitored sources are individual shallow wells, likely constructed to tap the phreatic or upper unconfined aquifer layers, typically at depths of less than 30 m. However, a subset of sites is presumed to access deeper, semi-confined aquifers through mechanically drilled wells, potentially exceeding 60 m in depth. Small-scale community supply systems, which likely fall within this deeper-well subset, were excluded from the analysis due to their differing management and treatment practices. Future work should prioritize incorporating well logs or direct stratigraphic data to improve vertical resolution and aquifer-specific assessments.

Information on the precise location of wells relative to potential pollution sources was not systematically available; however, for most monitored units, legally established sanitary protection zones apply, which substantially limit the influence of nearby activities on groundwater quality, in accordance with Romanian legislation (Government Decision No. 930/2005) [44].

As this study constitutes a secondary analysis of routine monitoring data, the temporal frequency and spatial distribution of samples were determined by user demand, not by a predefined probabilistic sampling design. This opportunistic sampling framework may lead to over-representation of sources with known or suspected water quality problems, while under-representing consistently compliant supplies. However, registry records indicate that over 90% of authorized groundwater supplies in Timiș County submitted at least one sample during the study period. Thus, the dataset also reflects broad participation among compliant systems and captures the practical realities of decentralized water quality monitoring across diverse meteorological conditions. Potential sampling bias is considered further in the Discussion section.

In addition, as the study relies on routine regulatory and voluntary monitoring rather than probabilistic sampling, it did not include microbial community–level analyses, which would require data and methodologies not uniformly available within the existing monitoring framework. Accordingly, the underlying data are not intended to represent population-level prevalence or long-term contamination trends, and the analyses focus on meteorological associations under real-world monitoring conditions rather than on estimating population trends.

To ensure temporal consistency in trend analyses, data from 2020 were excluded, due to the known disruptions in monitoring activities related to the COVID-19 pandemic. However, individual 2020 samples were retained for inclusion in correlation analyses where available.

## 3. Results

### 3.1. Microbiological Water Quality Analysis

Among well samples tested for HPC 22, 98.7% were below detection, with only 1.3% exceeding the defined microbiological threshold. By comparison, 6.97% of HPC 22 samples from the distribution network (140 of 2010) exceeded the acceptable limit. A similar pattern was observed for HPC 37, with exceedance rates of 2.31% in well samples and 11.0% in network samples. Coliform bacteria were detected above acceptable levels in 10.6% of well samples compared to 7.69% of network samples. *E. coli* showed the same directional trend: 4.85% of well samples were positive, compared to 2.31% in network samples (Table 2).

The type of water supply was significantly associated with the risk of microbial non-conformities, as shown by univariate logistic regression analyses (Table 3). Compared to water from the surface networks, water from groundwater wells had substantially lower odds of exhibiting high levels of general microbial activity, both at 22 °C (HPC 22: OR = 0.18, 95% CI: 0.14–0.23) and 37 °C (HPC 37: OR = 0.19, 95% CI: 0.15–0.24). However, well water was more likely to be contaminated with coliform bacteria (OR = 1.31, 95% CI: 1.15–1.49) and *E. coli* (OR = 2.07, 95% CI: 1.68–2.57). Conversely, network-supplied water showed higher odds of exceeding limits for heterotrophic plate count (HPC 22: OR = 5.84; HPC 37: OR = 5.59) but was less likely to be contaminated with coliforms (OR = 0.76, 95% CI: 0.66–0.86) and *E. coli* (OR = 0.51, 95% CI: 0.41–0.63).

### 3.2. Meteorological Data

Descriptive statistics for this data are presented in Table 4. The study period featured a wide temperature range (median 13.1 °C, IQR: 6–20.6 °C), with frequent high temperatures. Precipitations were sparse on most days (median 0 mm), but occasional heavy rain events (high IQR) were noted. Wind speed was moderate (median 6 m/s), but some exceedances were noted. Insolation varied seasonally with a median of 6.3 h/day.

### 3.3. Meteorological Influence on Water Quality

#### 3.3.1. Univariate Analysis

Univariate logistic regression assessed the relationship between individual meteorological factors (temperature, precipitation, wind speed, and insolation) and the presence of microbial non-conformities (HPC 22, HPC 37, coliforms, and *E. coli*) at lag times from 0 to 30 days (Table 5).

Meteorological variables had significant but distinct effects on microbial non-conformities in groundwater wells and network-supplied water.

In groundwater wells, higher average temperatures (especially 2–3 days prior) were associated with increased odds of HPC, coliforms, and *E. coli* detections. Short-term precipitation (2–3 days) increased the odds of HPC exceedances, while cumulative 30-day rainfall showed a protective effect. Higher wind speed on the sampling day reduced the odds of HPC exceedances (HPC 22: OR = 0.83; HPC 37: OR = 0.86). Insolation over 30 days was associated with increased odds of contamination across all indicators.

In network systems, increasing temperatures up to 30 days prior elevated the risk of all microbial indicators. Short-term precipitation (3 days) increased the odds of HPC and coliform presence. Higher wind speeds on the sampling day were slightly protective against *E. coli* contamination (OR = 0.897). Increased 30-day insolation was positively associated with all microbial exceedances (e.g., HPC 22: OR = 1.064; *E. coli*: OR = 1.040).

#### 3.3.2. Multivariate Analysis

A LASSO Logistic Regression was applied to assess how various meteorological variables—namely temperature, precipitation, wind speed, and insolation—at specific time lags (Day 0, Days 1–3, and Day 30) influence the likelihood of bacterial exceedances. The full model included each parameter across these selected time points and can be expressed as follows:

**Outcome**  *~Temperature (Day 0 + Day 1 + Day 2 + Day 3 + Day 30) + Precipitations (Day 0 + Day 1 + Day 2 + Day 3 + Day 30) + Wind speed (Day 0 + Day 1 + Day 2 + Day 3 + Day 30) + Insolation (Day 0 + Day 1 + Day 2 + Day 3 + Day 30).*

Cross-validation was employed to select the regularization parameter (λ), aiming to balance model flexibility with interpretability. For groundwater wells, the λ_1se_ criterion (one standard error above the minimum cross-validated error) was chosen to favour model sparsity and parsimony. In contrast, for distribution networks, the λ_min_ value (corresponding to the minimum cross-validated error) was used, as λ_1se_ led to over-penalization and the exclusion of relevant predictors.

In groundwater wells, exceedance of microbial water quality standards was primarily associated with precipitation, wind speed, and insolation, as shown in Table 6. For HPC at 22 °C, rainfall 2–3 days prior (ORs = 1.06 and 1.08) significantly increased the odds of exceedance, while wind speed on the same day and 2 days prior showed a protective effect (ORs = 0.83 and 0.85). Insolation 2 days prior was also protective (OR = 0.90), whereas insolation 30 days earlier increased the risk (OR = 1.12). For HPC at 37 °C, a similar pattern emerged: wind speed on the same day and 2 days prior was protective (ORs = 0.86 and 0.85), while insolation 1 and 30 days prior increased the odds (ORs = 0.92 and 1.11). Interestingly, precipitation 1 day prior had a mild protective effect (OR = 0.94). Coliform exceedances were driven by increased temperature 1 and 30 days prior (ORs = 1.03 each), while *E. coli* presence was strongly associated with same-day average temperature (OR = 1.07).

In piped network systems, microbial exceedances were influenced primarily by temperature and precipitation (Table 7). For HPC at 22 °C, increased temperature 30 days earlier (OR = 1.06) and rainfall on the current day and 3 days prior (ORs = 0.92 and 1.04) were relevant factors. HPC at 37 °C was similarly associated with precipitation and delayed temperature effects (ORs = 1.03 for both variables at 30 days). Coliform levels were linked to cumulative precipitation (Day 3 and Day 30; ORs = 1.03 and 1.05), while wind speed 30 days prior appeared protective (OR = 0.94). For *E. coli*, recent and delayed rainfall (Days 1 and 30) increased risk (ORs = 1.07 and 1.06), while same-day wind speed was associated with a lower likelihood of exceedance (OR = 0.88).

### 3.4. Seasonal Dynamics of Microbial Contamination and Associated Meteorological Drivers

The dataset revealed distinct seasonal variations in water quality correlated with temperature and precipitations.

**Figure 3:**
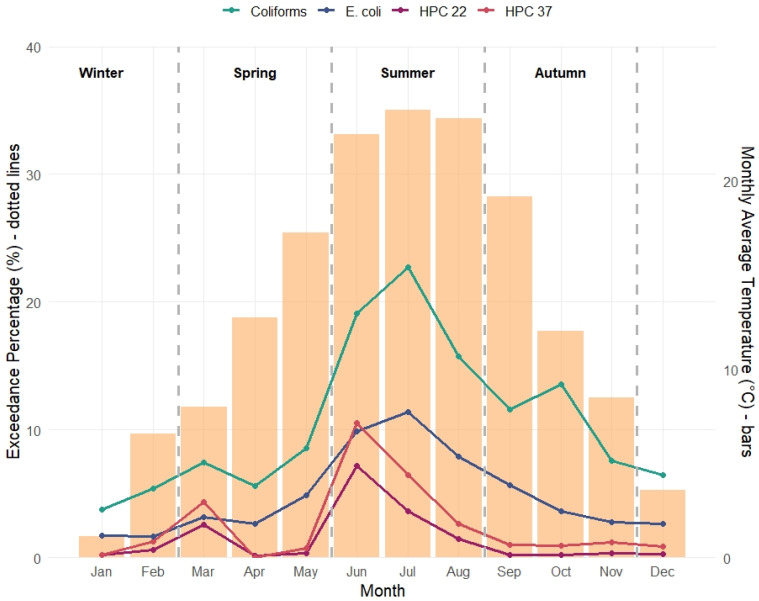
Monthly variation in Well water contamination and corresponding Average Temperature; coliform seasonality was statistically significant (Kruskal–Wallis: χ^2^ = 9.15, *p* = 0.027).

Exceedance of microbial water quality standards showed a clear pattern aligned with rising temperatures, as shown in Figure 3. A Kruskal–Wallis test confirmed significant seasonal differences in coliform exceedance rates in groundwater wells (χ^2^ = 9.15, *p* = 0.027), supporting the presence of seasonality. HCP 22, HCP 37, and coliforms increased progressively from spring, peaking in June–July, concurrent with the highest monthly average temperatures. *E. coli* levels remained lower than coliforms overall but exhibited a similar rise during the same periods. As temperatures declined from late summer into autumn, exceedance rates also decreased. These findings suggest a strong positive correlation between temperature and microbial contamination, both for heterotrophic plate counts and coliforms. These findings highlight a seasonal pattern in microbial non-conformities, consistent with higher temperatures and environmental loading in warmer months.

**Figure 4:**
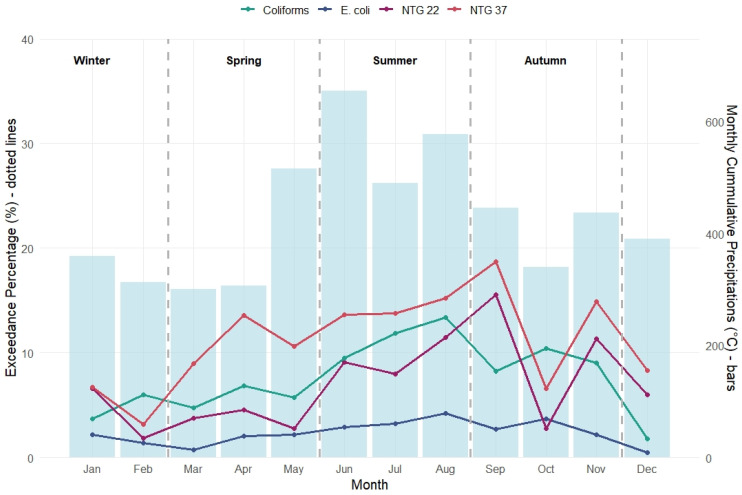
Monthly variation in Network water contamination and corresponding Cumulative Precipitations; HPC 37 (same as NTG 37) seasonality was not statistically significant (Kruskal–Wallis: χ^2^ = 6.39, *p* = 0.094).

Monthly cumulative precipitation peaked in May–June and again in August, but microbial exceedances followed with a delay of 1–2 months, suggesting a lagged effect of rainfall on contamination risk (Figure 4). More precisely, the highest exceedance rates for coliforms, HPC 22, and HPC 37 were recorded between June and September, peaking in September, and were preceded by sustained periods of elevated rainfall during the summer months. Notably, *E. coli* exceedance remained consistently low throughout the year, reflecting the generally higher microbial integrity of network-supplied water compared to wells from this perspective. Among all indicators, HPC 37 consistently displayed the highest seasonal variability, underscoring its value as a sensitive marker of meteorological influence on microbial water quality in piped distribution network; however, formal testing did not confirm statistically significant seasonal differences (Kruskal–Wallis χ^2^ = 6.39, *p* = 0.094). This suggests that HPC 37 dynamics in piped distribution networks are more indicative of short-term reactivity to meteorological forcing rather than a stable, recurring seasonal pattern.

### 3.5. Time Series Analysis: ARIMAX Modelling with Meteorological Covariates–Monthly Versus Weekly Performance

To investigate temporal dependencies and improve predictive accuracy, Auto-Regressive Integrated Moving Average models with exogenous variables (ARIMAX) were developed for microbial exceedance time series in both groundwater wells and piped distribution networks. Models were fitted at two temporal resolutions—monthly and weekly—to compare the influence of aggregation scale on forecast accuracy and residual adequacy. The approach aimed to (1) identify autocorrelation patterns and seasonal dynamics in microbial indicator exceedances, (2) quantify the predictive role of key meteorological variables (average temperature, precipitation, wind speed, and insolation) at relevant lags, and (3) determine the optimal model configuration for each indicator and water source type based on error metrics and Ljung–Box residual diagnostics.

The ARIMAX modelling results (presented in Table 8) indicated differences in predictive performance across source types, indicators, and temporal resolutions. For groundwater wells, monthly models consistently outperformed weekly models in terms of accuracy and residual adequacy, with precipitation at a 2-day lag emerging as a significant predictor for both HPC 22 and HPC 37. Coliform exceedances were best explained by average temperature 30 days prior, while *E. coli* was strongly associated with same-day temperature. In contrast, piped distribution networks showed weaker meteorological signal capture, with most models failing to identify consistent predictors. Notably, only *E. coli* in weekly models retained a significant association with average temperature, while other indicators lacked robust covariates.

Across all comparisons, the monthly groundwater well models provided the best fit (e.g., HPC 22 with RMSE = 6.80, MAE = 4.34, and Ljung–Box *p* = 0.934), suggesting that aggregated time scales enhance the ability to detect lagged meteorological influences, particularly in decentralized well systems.

**Figure 5:**
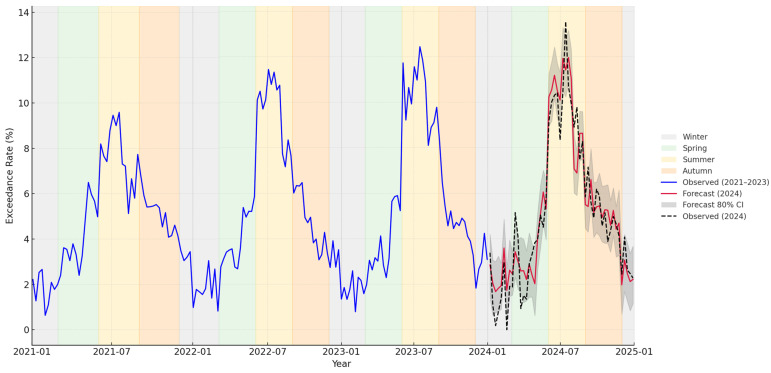
Weekly *E. coli* bacteria exceedance rates in groundwater wells for 2021–2023 with ARIMAX-model based projection for 2024, using actual 2024 meteorological data. While a de facto projection would typically rely on predicted meteorological inputs from weather forecasts, this approach uses real (observed) weather conditions from 2024, allowing for direct and more meaningful comparison between the model projection and the measured exceedance data. Observed exceedance rates were smoothed using a centred moving average filter with a 3-week window. This procedure reduced high-frequency noise and short-term spikes, emphasizing the underlying seasonal trend. Importantly, the ARIMAX model was fitted to raw weekly data, with smoothing applied only for visualization and comparability.

The projection for 2024 presented in Figure 5, based on the weekly ARIMAX model incorporating average temperature as an exogenous predictor, successfully reproduces the real seasonal pattern and peak timing of *E. coli* exceedances in groundwater wells. The model closely tracks observed 2024 values, particularly around the July–August maximum (~13%), indicating its ability to capture short-term variability as well as broader seasonal trends. Slight underestimation occurs in early spring and late summer, while some overestimation appears in late spring and early summer, which may be due to unmodeled short-lived contamination episodes or operational changes.

## 4. Discussion

### 4.1. Principal Findings and Their Implications

This comprehensive analysis of 15,394 microbiological test results from Timiș County, Western Romania, provides evidence for significant meteorological influences on drinking water quality across different supply systems. The observed patterns reveal distinct microbial contamination profiles between groundwater sources and the public distribution network, underscoring the importance of tailored monitoring and intervention strategies.

In our study, indicators of general microbial growth (HPC 22 and HPC 37) more frequently exceeded acceptable levels in the distribution network compared to groundwater sources, as observed consistently across seasonal summaries and regression analyses (Table 5, Table 6 and Table 7, Figure 3 and Figure 4). This may be attributed to conditions favourable to bacterial regrowth, such as biofilm formation, extended residence time, and suboptimal residual disinfectant levels especially at end points, particularly in older or poorly maintained systems [45,46]. Importantly, this difference concerns baseline microbial abundance rather than variability, as groundwater wells, despite generally lower HPC levels, may exhibit greater temporal fluctuations in response to meteorological forcing.

Consistent with our observations in piped networks, a study published by Edberg et al. (2005) documented significant increases in HPC levels within distribution systems compared to water at the source, emphasizing the role of infrastructure in facilitating microbial proliferation during transport [6]. Gilpin et al. (2024) conducted a comprehensive review of heterotrophic plate count (HPC) monitoring in distribution systems and confirmed that microbial regrowth is a recurrent issue due to biofilm formation, nutrient availability, and residual chlorine decay within pipes [47]. Similarly, Faraji et al. (2025) examined rural distribution systems and found that elevated ambient temperatures, increased turbidity, and reduced chlorine levels were significantly associated with higher microbial loads, even in well-treated water [48]. Waegenaar et al. (2024), through a pilot-scale study, further demonstrated that both water source and environmental temperature significantly influence bacterial regrowth, with treated groundwater showing increased HPC levels under warm conditions [49].

Conversely, our study results show that microbial exceedance indicators (*E. coli* and coliform bacteria) were more frequently detected in well water samples. Although legally established sanitary protection zones are in place for most monitored wells, these measures may not fully eliminate the risk of episodic contamination, particularly under conditions of intense precipitation or inadequate maintenance. In this context, exceedances are likely related to source-level vulnerabilities, such as shallow well construction, incomplete aquifer isolation, or localized environmental exposure to agricultural runoff and domestic wastewater. These findings are consistent with existing literature highlighting the vulnerability of unprotected or poorly maintained wells to microbial contamination, especially in rural and peri-urban contexts [50,51].

These findings align with previous research documenting the vulnerability of groundwater sources to fecal contamination. For example, a study conducted in India, Onda et al. (2012) found that shallow drilled wells (tubewells) often lacked sanitary sealing, leading to elevated levels of faecal indicator bacteria, particularly during the rainy season [52]. Additionally, a global review by Bain et al. (2014) confirmed that even “improved” water sources, including protected wells, frequently contain faecal contamination—especially in low-resource settings [53].

The temporal lag analysis represents a methodological advancement over previous studies that typically examined only concurrent relationships. Our systematic evaluation of 0-day, 1–3 day, and 30-day lag effects revealed that precipitation impacts on wells manifest within 2–3 days (OR = 1.06–1.08 for HPC exceedances), as quantified in the LASSO logistic regression models (Table 6 and Table 7). This is consistent with rapid infiltration through shallow soil layers and maybe even compromised wellhead protection. In contrast, network systems showed more prolonged temperature effects extending to 30 days (OR = 1.06 for HPC 22), possibly reflecting cumulative stress on distribution infrastructure and biofilm dynamics, as suggested by Tornevi et al. (2014) [54].

### 4.2. Mechanisms Underlying Meteorological Impacts on Water Quality

#### 4.2.1. Temperature-Driven Microbial Dynamics

The consistent positive correlation between temperature and all bacterial indicators (*p* < 0.001) aligns with established microbial ecology principles. Higher temperatures accelerate both bacterial multiplication rates, reduce die-off of vegetative pathogens, and enhance biofilm formation within distribution systems. The seasonal analysis performed in our study confirmed peak contamination rates during June-July, coinciding with maximum temperatures, supporting the temperature-growth relationship across both source types.

The exponential relationship between temperature and bacterial contamination aligns with fundamental microbiological principles and previous studies [55,56]. According to Spinoni et al. (2015), the frequency and severity of temperature extremes and drought events are projected to increase across Europe under moderate to high emission scenarios, with Southern and Eastern Europe particularly vulnerable [57]. These climatic trends may indirectly exacerbate microbiological risks in water systems by extending periods of elevated temperatures and low flow conditions [57].

For *E. coli* specifically, the strong association with same-day temperature in wells (OR =1.07, 95% CI: 1.06–1.08) could imply that thermal stress on pre-existing microbiological contamination facilitates rapid mobilization and detection [58]. This finding has practical implications for sampling protocols, as risk-based monitoring should intensify during heat waves and sustained warm periods.

#### 4.2.2. Precipitation-Mediated Contamination Pathways

In our study, short-term rainfall (2–3 days) increased contamination risk for all microbiological parameters in wells. This pattern is consistent with rapid surface–subsurface transfer processes, whereby intense or recent precipitation facilitates the infiltration of contaminated runoff, mobilizes fecal material and microorganisms stored in the unsaturated zone, and promotes preferential flow through soil macropores or compromised wellhead protection, as described by Lapworth et al. (2012) [27].

Conversely, prolonged cumulative precipitation (30 days) showed protective effects in wells for HPC 22 (OR = 0.85, CI: 0.72–0.96) and HPC 37 (OR = 0.81, CI: 0.70–0.91), likely reflecting dilution of contaminant concentrations, increased groundwater recharge, and flushing of previously accumulated surface-derived microbial loads, consistent with hydrological mechanisms during sustained wet periods emphasized by Santiago-Rodriguez et al. [59].

In our study, network systems exhibited delayed precipitation effects for coliform exceedances, with a significant association observed at a 3-day lag (OR = 1.03, CI: 1.01–1.05). This pattern suggests infiltration into aging distribution infrastructure, particularly in peri-urban areas where pipe integrity may be compromised. The seasonal pattern showing peak exceedances 1–2 months after maximum rainfall further supports this interpretation, as repeated wet–dry cycles can progressively weaken pipe materials and create delayed intrusion pathways. Similar mechanisms of contamination through pressure-driven intrusion in intermittent and deteriorated networks have been documented in peri-urban settings by Renwick et al. in 2013 [60], while a recent climate-impact assessment by Fan et al. (2023) have linked precipitation variability and wet–dry cycling to increased pipe failure probabilities in water supply systems [61].

The complex relationship between precipitation and water quality reflects multiple competing mechanisms. Heavy rainfall events are widely recognized to elevate the risk of water contamination. While literature reports increase ranging from modest to substantial, the precise magnitude depends heavily on regional characteristics—often seen as 2–3× in well-developed temperate regions, and potentially higher in tropical or low infrastructure contexts [62,63].

#### 4.2.3. Wind and Insolation Effects

The protective effect of higher wind speeds on same-day contamination for HPCs (HPC 22: OR = 0.83, HPC 37: OR = 0.86) and 1-day lag for coliforms and *E. coli* (OR = 0.90) may reflect enhanced atmospheric dispersion of surface contaminants and reduced settling of airborne particles near water sources. In contrast, the positive association between 30-day cumulative insolation and microbial exceedance risk (OR = 1.06 for HPC and OR = 1.05 for coliforms) indicates a delayed relationship with sustained warm and sunny conditions. While the precise mechanisms cannot be directly confirmed in this study, prolonged periods of high insolation may create environmental conditions favourable to microbial persistence or regrowth, such as increased water temperature or biofilm activity, as previously mentioned by other studies like Xiao et al. in 2024 [64].

### 4.3. Predictive Modelling for Early Warning Using ARIMAX

Predictive modelling represents a distinct methodological need, warranting separate discussion apart from regulatory considerations. Firstly, the ARIMAX models developed in this study underline the greater meteorological sensitivity of groundwater sources compared to piped networks, as well as the value of monthly aggregation for improving predictive robustness.

For *E. coli*, the monthly ARIMAX models achieved MAE values of 1.53–2.26 percentage points. Given the low average exceedance rate of 4.85% across the year, these deviations translate into relative errors of approximately 32–47% when expressed as mean absolute percentage error (MAPE). The apparent inflation of MAPE values reflects the mathematical sensitivity of percentage-based error metrics when observed rates are small, rather than poor model performance [65]. Importantly, during summer months when exceedance rates reach 12–13%, the same absolute deviations correspond to relative errors of only ≈12–17%, underscoring that MAE provides a more stable and interpretable assessment of predictive accuracy in this context. More broadly, the predictive errors observed across all microbial indicators (MAE ≈ 2–10 percentage points; RMSE ≈ 3–14) are consistent with, and in some cases lower than, values reported in comparable microbial water quality forecasting studies [63,66,67,68], reinforcing the robustness of the modelling approach.

Moreover, the strong correspondence between fitted and observed series, along with satisfactory residual diagnostics, underscores the utility of the weekly ARIMAX framework for near-real-time monitoring when actual meteorological inputs are available, like accurate forecasts. However, the model slightly underestimated summer 2024 exceedance magnitudes and overestimated some short spikes—likely due to local, non-meteorological disturbances (infrastructure works, point-source events). These limitations are expected in an operational setting and can be mitigated by (i) refreshing model fits seasonally, (ii) adding infrastructure/operational covariates (e.g., planned repairs, pressure anomalies), and (iii) pairing ARIMAX with anomaly detectors on residuals to flag non-weather shocks [69]. Nonetheless, the overall alignment between the timing of projected and observed peaks highlights the model’s utility as a predictive early warning tool. By reliably identifying high-risk periods—even if not always matching exact magnitudes—the model supports targeted, seasonal intervention planning, including preventive monitoring, public advisories, and operational adjustments. Its capacity to generalize spike behaviour under real weather inputs strengthens its relevance for proactive and climate-resilient drinking water management [70].

It is important to note that the ARIMAX models developed in this study are intended as exploratory and operational tools rather than fully optimized forecasting frameworks. Limitations include the use of aggregated weekly and monthly data, the absence of formal time-series cross-validation and stationarity testing, and the exclusion of non-meteorological drivers such as infrastructure disturbances or operational interventions. Consequently, ARIMAX results should be interpreted primarily in terms of temporal sensitivity and early-warning potential rather than precise prediction of exceedance magnitudes.

### 4.4. Emerging Approaches for Advanced Forecasting and Risk Modelling: Machine Learning and Probabilistic Models

Beyond ARIMAX, several advanced modelling tools offer promising capabilities for enhancing water-quality forecasting. These tools can accommodate non-linear dynamics, seasonal variability, and complex covariate relationships more effectively than traditional methods.

Tree-Based Ensembles such as Random Forest, Gradient Boosting, and XGBoost excel in handling non-linear interactions among predictors and are robust to multicollinearity—common in environmental datasets. For instance, an ensemble gradient boosting approach achieved over 93% accuracy in water potability classification, outperforming baseline models in an urban context [71]. Another study reported that in WQI forecasting, Random Forest and XGBoost outperformed other techniques with high discriminative power [72].

Generalized Additive Models (GAMs) enable flexible modelling via smooth functions, making them well-suited to capture complex, non-linear effects of hydrological and meteorological variables on water quality. They offer interpretability and can effectively visualize dependencies—such as rainfall impacts on turbidity or streamflow effects on WQI [14].

Deep learning architectures, including Long Short-Term Memory (LSTM) and Gated Recurrent Unit (GRU) networks are powerful tools for capturing long-range temporal patterns in water-quality parameters using sequential data. In one recent study, LSTM models achieved strong predictive performance (R^2^ between 0.79 and 0.86) in forecasting water quality indices such as total nitrogen and dissolved oxygen across rivers [73]. Recent applications in river and groundwater monitoring demonstrated that LSTM models achieved high predictive performance for microbial and chemical indicators, often surpassing traditional ARIMA-based approaches [74].

In addition, Wei et al. (2024) showed that Random Forest and LSTM models can forecast free chlorine residual in building water systems using in-line sensor data (ORP, pH, conductivity, temperature) combined with Wi-Fi-based occupancy proxies. The models provided accurate short-horizon predictions (≤5 min; in some cases up to 24 h), supporting proactive “sense–analyse–decide” management [75]. The application of artificial intelligence, including machine learning, is increasingly recognized globally for managing and predicting water quality issues in diverse water supply systems, necessitating the development of real-time monitoring and extensive data for robust model development [69,76,77].

Hybrid and probabilistic frameworks also hold potential. Hybrid ARIMA–machine learning models combine the strengths of linear and non-linear modelling, while probabilistic forecasting tools such as quantile regression, Bayesian state-space models, or conformal prediction provide exceedance probabilities instead of single-point forecasts, better aligning with operational risk management needs [71,78].

Importantly, many of these advanced and probabilistic approaches explicitly address uncertainty and variability through ensemble dispersion, probabilistic outputs, or Bayesian inference. In the context of highly variable microbial monitoring data, such methods could complement deterministic models by providing uncertainty bounds or exceedance probabilities rather than single-point predictions. While the present study focused on interpretable, parsimonious models compatible with routine monitoring data, future work integrating probabilistic or machine-learning–based frameworks could offer a more explicit quantification of uncertainty and support risk-based decision-making.

### 4.5. Public Health and Regulatory Implications

#### 4.5.1. Implications for Surveillance and Regulation

The higher microbial exceedance rates in wells (*E. coli* positive: 4.85%) compared with networks (2.31%) underscore persistent challenges in decentralized supplies. While both rates exceed WHO guidelines for treated water (zero tolerance), the well contamination levels are particularly concerning given the reliance of many rural communities on untreated or minimally treated groundwater.

The seasonal predictability of contamination peaks (June–September) creates opportunities for targeted, cost-effective action: (i) enhanced surveillance and sanitary inspections for private and small public wells during high-risk months; (ii) preventive advisories to well users (boil water, point-of-use chlorination, safe storage); (iii) utility-level operational adjustments in piped systems (maintaining disinfectant residuals, flushing of low-turnover zones, pressure management in areas prone to intrusion) [79].

In addition to traditional public health interventions, model-informed strategies offer complementary benefits. Such approaches can enable pre-emptive operational measures, including residual disinfectant boosting, targeted flushing, and intensified sampling ahead of forecasted high-risk windows. They also support timely public communication through advisories issued when exceedance probabilities surpass action thresholds, and resource prioritization by directing field teams to the sources or districts with the highest predicted risk [70,80,81,82,83].

#### 4.5.2. Adaptation Strategies and Operationalization

Climate-informed management actions—distinct from the immediate, seasonal operational interventions—should prioritize medium- to long-term system hardening and anticipatory control: (i) engineering protection of wells (wellhead sealing, surface drainage, setback enforcement), with attention to heavy-rainfall periods and regions; (ii) infrastructure resilience in piped distribution networks (pipe renewal in intrusion-prone districts, pressure zone optimization, backflow protection); (iii) climate-informed monitoring protocols that formalize lag-based trigger thresholds and adjust sampling intensity by forecasted risk; and (iv) early-warning systems that integrate meteorological feeds with water-quality models to enable pre-emptive interventions [22].

The differential vulnerability patterns we identified suggest that adaptation strategies must be tailored by source type. Wells require protection-focused interventions (improved wellhead sealing, drainage improvements, sanitary setbacks) [52], while networks need operational adaptations (enhanced disinfection during warm periods, pressure management during rainfall events, biofilm control strategies) [22].

The implementation of real-time, IoT-based water quality monitoring systems has significantly advanced the capacity to detect early signs of contamination, enabling rapid and targeted interventions that reduce the likelihood of disease outbreaks and infrastructure strain [84]. These systems, supported by open-source and cost-effective sensor kits, are especially effective when integrated with adaptive sampling protocols that respond dynamically to meteorological triggers [85,86]. Community-based surveillance and smart field kits further enhance coverage in rural or vulnerable regions, empowering local response mechanisms and filling gaps left by centralized monitoring [85,87].

### 4.6. Study Limitations and Future Research Directions

This study shares two key constraints already outlined in the Data Limitations Section: the opportunistic nature of the monitoring dataset and the absence of well depth information, which precluded aquifer-specific stratification.

Additional limitations include the temporal resolution of daily meteorological data, which may miss short-term extreme events (e.g., hourly rainfall intensities) that could drive acute contamination episodes. Higher-resolution weather data combined with continuous water quality monitoring could reveal additional mechanistic insights.

Expanding the analytical framework to include chemical parameters (turbidity, pH, dissolved oxygen) would provide a more comprehensive understanding of weather-driven water quality dynamics. Additionally, incorporating pathogen-specific indicators beyond *E. coli* and coliforms could improve risk assessment capabilities.

### 4.7. Methodological Contributions and Transferability

The methodological approach developed in this study offers several innovations applicable to similar monitoring systems globally. The systematic lag analysis framework provides a template for quantifying delayed environmental responses that are often overlooked in cross-sectional studies. The integration of LASSO regression for parsimonious variable selection in high-dimensional environmental datasets addresses common challenges in water quality epidemiology. The successful application of ARIMAX modelling to predict microbial exceedances using meteorological covariates at weekly and monthly scales demonstrates the feasibility of operationalizing weather-based early warning systems. The model validation approach using actual rather than predicted meteorological data provides a robust framework for assessing predictive performance under real-world conditions.

These methods could be readily adapted to other temperate regions with similar climate patterns and water supply configurations. The analytical pipeline developed could serve as a foundation for establishing climate-responsive water quality monitoring programs in data-rich environments.

The documented meteorological sensitivity of water quality systems has profound implications under projected climate change scenarios for Southeastern Europe. Regional climate models predict increased temperature extremes, altered precipitation patterns, and more frequent extreme weather events—all factors that our analysis shows directly impact microbial water quality [57,88].

## 5. Conclusions

Our study indicates that, while climate factors influence both systems, the vulnerability profiles differ fundamentally: groundwater wells exhibit greater susceptibility to acute microbial exceedances (e.g., *E. coli*) following precipitation events, whereas piped distribution networks show higher vulnerability to heterotrophic regrowth driven by seasonal temperature increases. These distinct patterns likely reflect broader dynamics in temperate continental regions, supporting a necessary shift from static, routine compliance checks to adaptive, risk-based surveillance.

The integration of ARIMAX modelling provided a better tool for quantifying these relationships in comparison with the predecessor approach with ARIMA, particularly useful for groundwater sources. The results demonstrate the potential of climate-informed early warning systems to anticipate contamination risks rather than merely reacting to them. This supports preventive policies rather reactionary approaches. By identifying high-risk windows—such as lag periods following heavy rainfall—water utilities and public health agencies can target interventions, optimize resource allocation, and issue timely public advisories. This approach is applicable to rural and drilling-reliant populations worldwide, where climate change escalates water safety challenges.

This study validates an analytical framework—featuring lag-aware inference, parsimonious predictor selection, and time-series forecasting—that is readily transferable to other regions with routine monitoring data. However, the study also highlights the need for data refinement. Future research should prioritize the integration of precise well-depth and hydrogeologic data to stratify aquifer vulnerability. Furthermore, moving beyond linear forecasting to complementary nonlinear and probabilistic models could further refine exceedance probability estimates, providing a more granular basis for operational decision-making in a changing climate.

## Figures and Tables

**Table 1:** Microbiological water quality parameters, regulatory limits, and analytical methods (per Law 458/2002 and Order 7/2023).

Parameter	Unit	Regulatory Limit	Standard Reference	Method Used
Heterotrophic plate count at 22 °C	CFU/mL	100	SR EN ISO 6222:2004 [43]	Pour plate method
Heterotrophic plate count at 37 °C	CFU/mL	20	SR EN ISO 6222:2004	Pour plate method
*Escherichia coli*	CFU/100 mL	0	SR EN ISO 9308-1:2014/A1:2017/A1:2020	Membrane filtration, Chromogenic substrate
Total coliform bacteria	CFU/100 mL	0	SR EN ISO 9308-1:2014/A1:2017/A1:2020	Membrane filtration, Chromogenic substrate

**Table 2:** Distribution of Microbiological Results and Regulatory Exceedances in Groundwater Wells and Networks.

Variable		Below Detection Range	Detectable Range	Above Detection Range	Total Exceedances
A. Groundwater Wells					
	HPC 22	5922 (96.42%)	156 (2.54%)	64 (1.04%)	82 (1.34%)
	HPC 37	5893 (96.67%)	114 (1.87%)	89 (1.46%)	141 (2.31%)
	Coliforms	8442 (89.40%)	793 (8.40%)	208 (2.20%)	1001 (10.60%)
	*E. coli*	9142 (95.20%)	381 (3.97%)	85 (0.88%)	466 (4.85%)
B. Piped Distribution Networks					
	HPC 22	1736 (86.37%)	179 (8.91%)	95 (4.73%)	140 (6.97%)
	HPC 37	1469 (86.72%)	110 (6.49%)	115 (6.79%)	186 (10.98%)
	Coliforms	4467 (92.31%)	318 (6.57%)	54 (1.12%)	372 (7.69%)
	*E. coli*	5200 (97.69%)	114 (2.14%)	9 (0.17%)	123 (2.31%)

**Table 3:** Impact of Water Supply Type on the Risk of Microbial Non-Conformities in Drinking Water.

Variable	OR (CI 95%)			
	HPC 22	HPC 37	Coliforms	*E. coli*
Groundwater Wells	0.18 (0.14–0.23)	0.19 (0.15–0.24)	1.31 (1.15–1.49)	2.07 (1.68–2.57)
Piped Networks	5.84 (4.39–7.85)	5.59 (4.42–7.10)	0.76 (0.66–0.86)	0.51 (0.41–0.63)

Note: All reported odds ratios are significant at *p* < 0.001.

**Table 4:** Meteorological Variables (2015–2024).

Variable	Mean ± St. Dev (N = 3275)	Median (IQR) (N = 3275)
Average temperature (°C)	13.08 ± 8.70	13.10 (6–20.60)
Maximum temperature (°C)	18.49 ± 10.18	18.90 (10.60–27.10)
Minimum temperature (°C)	7.67 ± 7.71	7.70 (1.30–14.40)
Precipitation (mm)	1.66 ± 4.49	0 (0–0.90)
Wind speed (m/s)	6.56 ± 2.76	6.00 (5.00–8.00)
Insolation (hours/day)	5.88 ± 4.07	6.30 (1.90–9.50)

**Table 5:** Impact of Meteorological Parameters on the Risk of Microbial Non-Conformities in Drinking Water.

Variable	Lag (Days)	OR (CI 95%)			
		HPC 22	HPC 37	Coliforms	*E. coli*
A. Groundwater Wells					
Average Temperature	0	1.035 (1.008–1.064) *	1.030 (1.009–1.052) **	1.056 (1.047–1.065) ***	1.068 (1.055–1.082) ***
	1	1.028 (1.000–1.057) *	1.032 (1.011–1.054) **	1.058 (1.049–1.067) ***	1.069 (1.056–1.083) ***
	2	1.040 (1.012–1.069) **	1.039 (1.018–1.061) ***	1.054 (1.045–1.063) ***	1.064 (1.051–1.077) ***
	3	1.056 (1.028–1.086) ***	1.046 (1.025–1.068) ***	1.054 (1.045–1.063) ***	1.064 (1.051–1.077) ***
	30	1.031 (1.005–1.058) *	1.034 (1.014–1.054) ***	1.052 (1.044–1.061) ***	1.059 (1.046–1.072) ***
Precipitations	0	1.015 (0.978–1.041)	1.000 (0.965–1.027)	1.009 (0.997–1.020)	1.009 (0.992–1.024)
	1	1.002 (0.946–1.047)	0.976 (0.926–1.018)	1.008 (0.994–1.022)	1.004 (0.983–1.023)
	2	1.078 (1.045–1.109) ***	1.045 (1.015–1.073) **	0.999 (0.985–1.013)	0.992 (0.969–1.013)
	3	1.058 (1.037–1.077) ***	1.027 (1.003–1.048) *	1.019 (1.008–1.029) ***	1.026 (1.012–1.040) ***
	30	0.850 (0.716–0.955) *	0.813 (0.701–0.908) **	1.013 (1.002–1.023) *	1.014 (0.998–1.028)
Wind speed	0	0.831 (0.741–0.922) ***	0.859 (0.790–0.927) ***	0.977 (0.952–1.003)	0.966 (0.930–1.003)
	1	0.967 (0.884–1.048)	0.940 (0.875–1.003)	0.900 (0.874–0.926) ***	0.895 (0.858–0.932) ***
	2	0.953 (0.875–1.028)	0.941 (0.882–1.000)	0.932 (0.908–0.957) ***	0.938 (0.904–0.973) ***
	3	1.011 (0.934–1.086)	1.044 (0.986–1.100)	0.983 (0.959–1.006)	0.982 (0.948–1.015)
	30	1.032 (0.957–1.106)	1.010 (0.950–1.068)	1.003 (0.979–1.026)	1.012 (0.979–1.045)
Insolation	0	0.985 (0.933–1.040)	0.994 (0.954–1.037)	1.068 (1.050–1.087) ***	1.084 (1.058–1.111) ***
	1	0.984 (0.931–1.038)	0.987 (0.946–1.029)	1.084 (1.066–1.103) ***	1.105 (1.078–1.133) ***
	2	0.937 (0.886–0.989) *	0.982 (0.942–1.022)	1.080 (1.062–1.098) ***	1.091 (1.065–1.118) ***
	3	0.977 (0.926–1.030)	0.976 (0.936–1.016)	1.067 (1.050–1.085) ***	1.074 (1.049–1.099) ***
	30	1.141 (1.079–1.208) ***	1.136 (1.089–1.186) ***	1.073 (1.055–1.090) ***	1.089 (1.064–1.116) ***
B. Piped Distribution Networks					
Average Temperature	0	1.022 (1.001–1.043) *	1.029 (1.010–1.048) **	1.029 (1.016–1.043) ***	1.026 (1.004–1.049) *
	1	1.019 (0.998–1.040)	1.025 (1.006–1.044) **	1.034 (1.021–1.048) ***	1.031 (1.009–1.054) **
	2	1.027 (1.006–1.048) *	1.034 (1.015–1.053) ***	1.035 (1.022–1.049) ***	1.035 (1.012–1.059) **
	3	1.029 (1.008–1.051) **	1.036 (1.017–1.056) ***	1.038 (1.024–1.052) ***	1.042 (1.019–1.067) ***
	30	1.055 (1.032–1.078) ***	1.044 (1.024–1.065) ***	1.041 (1.027–1.055) ***	1.046 (1.023–1.072) ***
Precipitations	0	0.928 (0.857–0.987) *	0.986 (0.937–1.029)	0.981 (0.948–1.012)	0.962 (0.898–1.015)
	1	1.011 (0.960–1.057)	0.995 (0.947–1.040)	1.013 (0.983–1.041)	1.051 (1.010–1.089) **
	2	1.016 (0.973–1.054)	0.999 (0.958–1.036)	1.036 (1.014–1.057) ***	1.047 (1.012–1.078) **
	3	1.049 (1.021–1.076) ***	1.037 (1.006–1.067) *	1.027 (1.007–1.047) **	1.001 (0.963–1.031)
	30	1.000 (0.954–1.038)	0.993 (0.949–1.031)	1.036 (1.014–1.057) ***	1.043 (1.007–1.074) **
Wind speed	0	0.946 (0.877–1.015)	0.955 (0.895–1.016)	0.949 (0.909–0.989) *	0.897 (0.826–0.968) **
	1	0.970 (0.907–1.033)	0.969 (0.913–1.025)	0.975 (0.937–1.014)	0.935 (0.865–1.003)
	2	0.997 (0.938–1.056)	1.032 (0.978–1.086)	0.973 (0.934–1.011)	0.968 (0.902–1.032)
	3	1.039 (0.988–1.089)	1.036 (0.990–1.081)	0.964 (0.924–1.004)	0.933 (0.863–1.001)
	30	0.975 (0.913–1.037)	0.975 (0.919–1.030)	0.967 (0.929–1.005)	0.928 (0.863–0.992) *
Insolation	0	1.030 (0.988–1.075)	1.031 (0.994–1.071)	1.027 (1.001–1.055) *	1.022 (0.978–1.069)
	1	1.014 (0.972–1.059)	1.027 (0.989–1.068)	1.030 (1.003–1.058) *	1.002 (0.958–1.050)
	2	1.001 (0.959–1.045)	1.008 (0.969–1.047)	1.019 (0.992–1.047)	1.009 (0.964–1.057)
	3	0.988 (0.947–1.031)	1.007 (0.970–1.046)	1.021 (0.993–1.049)	1.018 (0.972–1.067)
	30	1.064 (1.018–1.113) **	1.062 (1.022–1.104) **	1.050 (1.021–1.079) ***	1.040 (0.992–1.090)

Note: * 0.01 ≤ *p* < 0.05, ** 0.001 ≤ *p* < 0.01, *** *p* < 0.001.

**Table 6:** LASSO Logistic Regression Results for Groundwater Wells: Statistically Significant Predictors of Microbial Exceedances at the Optimal λ_1se_ Penalty.

Outcome|Covariate	OR (CI 95%)	*p*-Value
Heterotrophic plate count at 22 °C–λ_1se_ = 0.00197, which yielded mean cross-validated deviance 0.150 (±0.013), Brier score 0.0166, and 10 nonzero coefficients		
Wind speed	0.83 (0.73–0.94)	0.003
Wind speed 2 days ago	0.85 (0.76–0.94)	0.002
Precipitations 2 day ago	1.06 (1.02–1.10)	0.003
Precipitations 3 days ago	1.08 (1.05–1.11)	<0.001
Insolation 2 days ago	0.90 (0.83–0.96)	0.003
Insolation 30 days ago	1.12 (1.05–1.21)	0.001
Heterotrophic plate count at 37 °C–λ_1se_ = 0.00327, which yielded mean cross-validated deviance 0.256 (±0.00881), Brier score 0.0295, and 13 nonzero coefficients		
Wind speed	0.86 (0.78–0.94)	<0.001
Wind speed 2 days ago	0.85 (0.77–0.93)	<0.001
Precipitations 1 day ago	0.94 (0.90–0.98)	0.012
Insolation 1 day ago	0.92 (0.86–0.99)	0.028
Insolation 30 days ago	1.11 (1.05–1.17)	<0.001
Coliforms–λ_1se_ = 0.0341, which yielded mean cross-validated deviance 0.696 (±0.0207), Brier score 0.0994, and 3 nonzero coefficients		
Average temperature 1 day ago	1.03 (1.01–1.06)	0.032
Average temperature 30 days ago	1.03 (1.02–1.04)	<0.001
*E. coli*–λ_1se_ = 0. 0272, which yielded mean cross-validated deviance 0.387 (±0.0224), Brier score 0.0459, and 1 nonzero coefficient		
Average temperature	1.07 (1.06–1.08)	<0.001

**Table 7:** LASSO Logistic Regression Results for Piped Distribution Networks: Statistically Significant Predictors of Microbial Exceedances at the Optimal λ_min_ Penalty.

Outcome|Covariate	OR (CI 95%)	*p*-Value
Heterotrophic plate count at 22 °C–λ_min_ = 0.0043, which yielded mean cross-validated deviance 0.554 (±0.023), Brier score 0.073, and 10 nonzero coefficients		
Average temperature 30 days ago	1.06 (1.02–1.10)	0.002
Precipitations current day	0.92 (0.85–0.98)	0.027
Precipitations 3 days ago	1.04 (1.01–1.07)	0.015
Heterotrophic plate count at 37 °C–λ_min_ = 0.0166, which yielded mean cross-validated deviance 0.767 (±0.0337), Brier score 0.1112, and 3 nonzero coefficients		
Average temperature 30 days ago	1.03 (1.01–1.06)	0.018
Precipitations 3 days ago	1.03 (1.01–1.06)	0.040
Coliforms–λ_min_ = 0.0317, which yielded mean cross-validated deviance 0.508 (±0.0253), Brier score 0.065, and 10 nonzero coefficients		
Precipitations 3 days ago	1.03 (1.01–1.06)	0.003
Precipitations 30 days ago	1.05 (1.03–1.07)	<0.001
Wind speed 30 days ago	0.94 (0.90–0.99)	0.022
*E. coli*–λ_min_ = 0.0013, which yielded mean cross-validated deviance 0.193 (±0.0258), Brier score 0.019, and 9 nonzero coefficients		
Precipitations 1 day ago	1.07 (1.02–1.12)	0.004
Precipitations 30 days ago	1.06 (1.03–1.09)	<0.001
Wind speed current day	0.88 (0.79–0.97)	0.013

**Table 8:** Comparison of Monthly and Weekly ARIMAX Models with Meteorological Covariates for Microbial Indicators in Groundwater Wells and Piped Networks.

Outcome	Frequency	ARIMA/ARIMAX Order	RMSE	MAE	Ljung–Box *p*	Significant Exogenous Predictors
A. Groundwater wells						
HPC 22	Monthly	(0,0,3)	6.80	4.34	0.934	Precipitations 2 day ago (*p* < 0.001)
	Weekly	(1,0,1)	10.58	6.34	0.942	none
HPC 37	Monthly	(2,0,0) (1,0,0) [s]	9.81	7.01	0.778	Precipitations 2 day ago (*p* < 0.001)
	Weekly	(0,1,1) (0,0,1) [s]	14.15	10.72	<0.001 *	none
Coliforms	Monthly	(1,0,1)	6.68	5.63	0.821	Avg. temp. 30 days ago (*p* = 0.008)
	Weekly	(0,0,2) (0,0,1) [s]	11.36	8.14	0.244	Avg. temp. 30 days ago (*p* = 0.035)
*E. coli*	Monthly	(0,0,0)	3.07	1.53	0.494	Average temperature (*p* < 0.001)
	Weekly	(0,0,0)	3.32	1.68	0.282	Average temperature (*p* < 0.001)
B. Piped Distribution Networks						
HPC 22	Monthly	(1,1,1)	11.93	7.18	0.224	none
	Weekly	(1,0,1)	9.55	6.02	0.942	none
HPC 37	Monthly	(1,0,0)	13.87	10.47	0.424	none
	Weekly	(0,1,1) (0,0,1) [s]	13.43	9.87	0.183	none
Coliforms	Monthly	(0,0,0)	5.94	4.14	0.578	none
	Weekly	(0,0,0)	11.36	8.14	0.244	Avg. temp. 30 days ago (*p* = 0.008)
*E. coli*	Monthly	(0,0,1)	3.34	2.26	0.011 *	none
	Weekly	(0,0,2) (0,0,1) [s]	7.16	3.97	0.084	Average temperature (*p* < 0.001)

Note: * Ljung–Box *p*-values ≤ 0.05 indicate inadequate residual diagnostics (non–white-noise residuals), suggesting remaining autocorrelation and possible model misspecification.

## Data Availability

The original contributions presented in the study are included in the article, further inquiries can be directed to the corresponding authors.

## Abbreviations

The following abbreviations are used in this manuscript:

ARIMA	Auto-Regressive Integrated Moving Average
ARIMAX	Auto-Regressive Integrated Moving Average with Exogenous Variables
CFU	Colony Forming Unit
CI	Confidence Interval
DEM	Digital Elevation Model
DSP	Public Health Directorate (Direcția de Sănătate Publică)
*E. coli*	*Escherichia coli*
EU	European Union
GAM	Generalized Additive Models
GRU	Gated Recurrent Unit
HPC	Heterotrophic Plate Count
IoT	Internet of Things
IQR	Interquartile Range
ISO	International Organization for Standardization
LASSO	Least Absolute Shrinkage and Selection Operator
LSTM	Long Short-Term Memory
MAE	Mean Absolute Error
MAPE	Mean Absolute Percentage Error
OR	Odds Ratio
RENAR	Romanian Accreditation Association (Asociația de Acreditare din România)
RMSE	Root Mean Squared Error
SRTM	Shuttle Radar Topography Mission
WHO	World Health Organization
WQI	Water Quality Index
